# Tabular Sheet of the American Society of Dental Surgeons

**Published:** 1849-04

**Authors:** 


### Miscellaneous Notices.

*Tabular Sheet of the American Society of Dental Surgeons.*—At the last meeting of the American Society of Dental Surgeons, Dr. C. O. Cone, submitted to the association a tabular sheet, so arranged, that with very little trouble, any dentist who keeps a correct record of all his cases, may present every fact connected with each one. In this way, if dentists generally would report their cases, and all the facts connected with each, a vast amount of valuable statistical information—information which would contribute in an eminent degree to the advancement of dental pathology and therapeutics, would be obtained; by collecting observations of this sort, the practitioner of dental surgery will not only be able to form a more correct opinion with regard to the probable result of operations upon teeth possessing different characteristics, and under all the varied circumstances in which their performances are called for. It would enable him, too, to determine, more correctly, the relative value of different therapeutic agents, and different methods of practice. The sheet is arranged with three tables, and in such a way that the age, sex, temperament, habit of body, and the characteristics of the teeth and other parts of the mouth, as well as the particular operation performed, and teeth operated on, may be exhibited at a single glance.

We hope that every dentist to whom this sheet has or may be sent, will furnish the result of his practice and observations of the past year, and forward it to Dr. Cone in time to prepare a report for the next meeting of the society, but if only twenty should do so, it would supply material for a highly interesting and valuable paper.

It is not sufficient for a practitioner to notice the failure of an operation upon the teeth, or any particular treatment or method of practice to which these organs, or their contiguous parts, have been subjected; it is important to know the cause, to ascertain whether it be wholly or in part the result of an inefficient application of the resources of our art, or to circumstances over which the operator has no control. In short, there are a thousand circumstances connected with the success and failure of dental operations, which should be fully understood and appreciated by every practitioner of dental surgery, and just in proportion as he becomes familiar with these will his usefulness and success be increased. It is not sufficient that a man be able to construct a dental substitute and even fill teeth in a skilful and neat manner, to be a good dentist. Dentistry is no longer a mere mechanical art that may be practiced by individuals wholly unacquainted with the laws of disease and health. Like all the other branches of medicine, it must be practiced upon scientific principles, and must be perfected by accumulating observations and facts, and it is for the purpose of doing this, that the tabular sheet in question has been issued. The getting of it up has been a work of much labor and study, and the admirable manner in which it is done, is certainly deserving of great credit.

It may be thought by some, that the sheet is too complex in its arrangement for any useful or practical purposes, and in consequence of which, few will be found who have kept a sufficiently accurate record of their cases, to enable them to supply the various items of information that it calls for. We cannot, however, regard this as a reasonable objection; it may be filled in whole or in part, according to the fullness of the record, which has been made of each individual case for the past year, or any given length of time. The fuller the report, of course the better, as more facts will thereby be elicited.

For the purpose of enabling dentists to make annual reports of the cases which may occur in their practice, or fall under their observation, we would recommend that each practitioner provide himself with a case book for the purpose. Several very excellent ones have, within the last two or three years, been prepared, one by Dr. H. Crane of Cincinnati, Ohio, another by J. G. Ambler of N. Y., and still another by Dr. Crane of Easton, Pa.

